# Cryoprecipitate transfusion in trauma patients attenuates hyperfibrinolysis and restores normal clot structure and stability: Results from a laboratory sub-study of the FEISTY trial

**DOI:** 10.1186/s13054-022-04167-x

**Published:** 2022-09-26

**Authors:** Gael B. Morrow, Timea Feller, Zoe McQuilten, Elizabeth Wake, Robert A. S. Ariëns, James Winearls, Nicola J. Mutch, Mike A. Laffan, Nicola Curry

**Affiliations:** 1 Nuffield Division of Clinical Laboratory Sciences, Radcliffe Department of Medicine, University of Oxford, Oxford, OX3 9DU UK; 2 Aberdeen Cardiovascular and Diabetes Centre, School of Medicine, Medical Sciences and Nutrition, Institute of Medical Sciences, University of Aberdeen, Aberdeen, UK; 3 Leeds Thrombosis Collective, Discovery and Translational Science Department, Leeds Institute of Cardiovascular and Metabolic Medicine, University of Leeds, Leeds, UK; 4 Transfusion Research Unit, Melbourne and Monash Health, Monash University, Melbourne, Australia; 5 Trauma Service, Gold Coast University Hospital, Southport, Australia; 6 School of Medicine and Dentistry, Griffith University, Gold Coast Campus, Southport, Australia; 7 Centre for Haematology, Imperial College London, London, UK; 8 Oxford Haemophilia and Thrombosis Centre, NIHR Oxford Biomedical Research Centre, Oxford University Hospitals NHS Foundation Trust, Oxford, UK

**Keywords:** Fibrinogen, Cryoprecipitate, Trauma coagulopathy, Fibrinolysis, Clot structure

## Abstract

**Background:**

Fibrinogen is the first coagulation protein to reach critical levels during traumatic haemorrhage. This laboratory study compares paired plasma samples pre- and post-fibrinogen replacement from the Fibrinogen Early In Severe Trauma studY (FEISTY; NCT02745041). FEISTY is the first randomised controlled trial to compare the time to administration of cryoprecipitate (cryo) and fibrinogen concentrate (Fg-C; Riastap) in trauma patients. This study will determine differences in clot strength and fibrinolytic stability within individuals and between treatment arms.

**Methods:**

Clot lysis, plasmin generation, atomic force microscopy and confocal microscopy were utilised to investigate clot strength and structure in FEISTY patient plasma.

**Results:**

Fibrinogen concentration was significantly increased post-transfusion in both groups. The rate of plasmin generation was reduced 1.5-fold post-transfusion of cryo but remained unchanged with Fg-C transfusion. Plasminogen activator inhibitor 1 activity and antigen levels and Factor XIII antigen were increased post-treatment with cryo, but not Fg-C. Confocal microscopy analysis of fibrin clots revealed that cryo transfusion restored fibrin structure similar to those observed in control clots. In contrast, clots remained porous with stunted fibres after infusion with Fg-C. Cryo but not Fg-C treatment increased individual fibre toughness and stiffness.

**Conclusions:**

In summary, our data indicate that cryo transfusion restores key fibrinolytic regulators and limits plasmin generation to form stronger clots in an ex vivo laboratory study. This is the first study to investigate differences in clot stability and structure between cryo and Fg-C and demonstrates that the additional factors in cryo allow formation of a stronger and more stable clot.

**Supplementary Information:**

The online version contains supplementary material available at 10.1186/s13054-022-04167-x.

## Background

Injury accounts for 9% of deaths globally or 4.9 million deaths every year [1], and is the leading cause of death in persons under the age of 44 [2]. Uncontrolled bleeding accounts for 25% of all injury-related deaths [3–10] and 40–80% of potentially preventable deaths [11]. Furthermore, death from traumatic haemorrhage is frequently early; 60% occur within the first 3 h of injury [12].

Over the past two decades the management of severe traumatic haemorrhage has evolved and has been driven by an increased understanding of the pathology of trauma-induced coagulopathy (TIC). TIC is a multi-phenotypic disease state that comprises disorders of coagulation and inflammation, and describes the overall failure of the coagulation system to maintain haemostasis after major injury. TIC is associated with significantly poorer outcomes, including increased need for massive transfusion and development of organ failure and a 3-fourfold increased risk of death [13–16].

Fibrinogen is the first coagulation protein to reach critically low levels during traumatic haemorrhage [17–19]. Fibrinogen is cleaved by thrombin to insoluble fibrin, which forms a haemostatic plug at sites of bleeding. Fibrin is a viscoelastic polymer and its properties are crucial in determining the physical and mechanical characteristics of the clot [20]. Fibrin cross-linking by the transglutaminase, activated factor XIII (FXIIIa), occurs between neighbouring fibrin molecules to enhance clot stability against mechanical stress [21, 22]. Alpha 2-antiplasmin (α_2_AP) is also cross-linked to fibrin via FXIII-A to stabilise the clot against premature degradation by plasmin [23, 24]. Current trauma guidelines suggest fibrinogen replacement should occur when plasma fibrinogen levels are below 1.5 g/L [25].

Hypofibrinogenaemia is associated with reduced clot strength, increased transfusion requirements and worse outcomes [19, 26]. Therefore, there is rationale for early fibrinogen replacement as an effective therapy for major trauma haemorrhage [27–29]. There are two main sources of fibrinogen replacement: cryoprecipitate and fibrinogen concentrate (Fg-C). Cryoprecipitate (cryo) is a pooled blood component derived from whole blood donations and has a variable but high fibrinogen concentration (8–16 g/L) [30]. Additionally, cryo is rich in a number of other coagulation factors that are not present in Fg-C [31]. These include anti-fibrinolytic factors such as plasminogen activator inhibitor 1 (PAI-1) and FXIII. Fg-C has been used for many years to prevent bleeding in inherited dysfibrinogenaemia and hypofibrinogenaemia, and has a favourable safety profile [32]. FEISTY trial utilises RiaStap (CSL Behring), which does not contain additional anti-fibrinolytic factors, however some commercially available Fg-C do contain FXIII [33, 34].

The Fibrinogen Early in Severe Trauma studY (FEISTY; NCT02745041) is the first randomised controlled trial (RCT) to compare the time to administration of cryo and fibrinogen Fg-C in trauma patients [35, 36]. This sub-study compares paired plasma samples pre- and post-fibrinogen supplementation from patients recruited to the FEISTY study and is the first study to investigate the impact of cryo and Fg-C treatment on fibrinolysis, clot structure and clot stability in a RCT setting. Previous in vitro work by the authors has shown that clots formed from cryo were more resistant to fibrinolytic degradation than those formed from Fg-C [37]. We hypothesised that the additional pro-coagulant and anti-fibrinolytic factors present in cryo may result in the formation of stronger, more stable clots when compared to Fg-C.

## Methods

### Eligibility criteria and randomisation

Full details of the FEISTY study are available [36]. Adult trauma patients (> 18 years) were eligible for the FEISTY study if they had clinically significant haemorrhage (either assessment of blood consumption score (ABC) score ≥ 2 or judged by treating clinicians to require massive transfusion) and a FIBTEM A5 result on admission of < 10 mm. FIBTEM A5 is the clot amplitude at 5 min after initial clot formation measured by rotational thromboelastography (ROTEM; Werfen, Barcelona, Spain). Ethical approval was granted by the Gold Coast Hospital and Health Service Human Research Ethics Committee (HREC/16/QGC/128). Patients were excluded if they had one of the following: injuries incompatible with survival, already received cryo or Fg-C or more than 6 h had elapsed between injury and presentation. Patients were randomly assigned to an intervention arm and subsequently received either Fg-C (Riastap, CSL Behrin GmbH, Marburg, Germany) or cryo (whole blood or apheresis cryo; Australian Red Cross Lifeblood) replacement. Fg-C and cryo dosing were guided by the FIBTEM A5 result and assuming that 1 g fibrinogen will result in an increment of 1–2 mm In the FIBTEM A5. Fibrinogen replacement was continued throughout resuscitation according to the FIBTEM A5 value. All baseline clinical data (Table 1) were obtained from the FEISTY investigators. Full details of the FEISTY study are available [36]. There was no research arm to the FEISTY trial and excess plasma samples were stored on an ad hoc basis which is reflected in the small number of patients in our laboratory study.

**Table 1:** Baseline clinical characteristics. Clinical information for patients in the cryoprecipitate and Fg-C cohorts including admission, pre-hospital, during hospital and injury demographics. Data is presented as median (IQR). 24 h blood product usage encompasses packed red blood cells (PRBC), fresh frozen plasma (FFP) and platelets

		Cryo	Fg-C
Patients	Number	9	13
	Age (year)	41 (27–61)	48 (41–61)
	Male, *n* (%)	9 (100)	4 (31)
Admission	Time to admission (min)	43 (28–110)	30 (12–126)
	Heart rate (beats/min)	120 (91–144)	130 (110–143)
	Systolic blood pressure (mmHg)	141 (115–148)	130 (114–145)
	Clauss fibrinogen (mg/ml)	2 (1.7–2.7)	1.9 (1.6–2.3)
	Platelets (× 10^9^ plt/L)	173 (155–251)	200.5 (125–238)
	Haemoglobin (g/L)	134 (101–146)	138 (126–158)
	PT (s)	16 (15–19)	16 (14–19)
	INR	1.3 (1.1–1.4)	1.2 (1.1–1.6)
	FIBTEM CA5 (mm)	9 (8.5–10)	7.5 (6.3–8.8)
	EXTEM CA5 (mm)	37 (30–40.5)	38 (32–39.5)
Injury	ISS	29 (20–35)	29 (18–33)
	GCS	15 (3–15)	3 (3–15)
	Blunt Injury, *n* (%)	8 (89)	10 (77)
	Multiple injuries, burns or other, *n* (%)	6 (67)	7 (54)
	Head and other associated injuries, *n* (%)	2 (22)	3 (23)
	Chest and/or abdominal injuries only, *n* (%)	1(11)	3 (23)
Pre-hospital	TXA, *n* patients (%)	2 (22.2)	7 (54)
	FFP, *n* patients (%)	0 (0)	1 (8)
	RBC, *n* patients (%)	4 (44)	7 (54)
In hospital	ICU length of stay (days)	8 (5–19)	5 (4–14)
	Hospital length of stay (days)	23 (17–45)	9 (4–45)
	Time to first dose (min)	112 (85–207)	48 (33–104)
	Time from first dose to sample (min)	74.5 (28–313)	25 (15–76)
	24 h blood products (units)	20 (19–38)	11 (7–20)
	Sepsis, *n* (%)	4 (44)	4 (31)
Arterial Thrombosis	Myocardial Infarction, *n* (%)	0 (0)	0 (0)
	Cerebrovascular Accident, *n* (%)	1 (11)	1 (8)
Venous Thrombosis	Deep vein thrombosis, *n* (%)	1 (11)	1 (8)
	Pulmonary Embolism, *n* (%)	3 (33)	0 (0)

### Blood collection

Investigations were conducted with the participants’ informed consent in agreement with the Declaration of Helsinki. Ethical approval was obtained from the Institutional Review Board of Oxford University Hospitals NHS Foundation Trust for analysis of the pre-collected trauma samples (18/EM/0056). FEISTY patient samples were transported from Australia on an excess of dry ice to the laboratory in Oxford. Plasma samples from 16 healthy volunteers and a pooled normal plasma (made in house from 20 healthy volunteers) were used as a control. All blood samples were collected in 0.13 M trisodium citrate vacutainers and platelet poor plasma was obtained by centrifugation of whole blood samples at 2500 g for 30 min at 4 °C. Plasma samples were stored at − 80 °C until analysis.

### Quantification of plasma protein concentrations

Concentrations of PAI-1, D-dimer, C-reactive protein (CRP) and urokinase plasminogen activator (uPA) were quantified using SimplePlex^™^ assays on the Ella™ system following the manufacturers’ instructions (Bio-Techne, Minnesota, USA). Fibrinogen, tissue type plasminogen activator (tPA), α_2_AP, thrombin activatable fibrinolysis inhibitor (TAFI), FXIII, thrombomodulin (TM) and syndecan-1 concentrations were determined using commercial Enzyme linked immunosorbent assays (ELISA) kits (Abcam, Cambridge, UK). Fibrinogen was also measured at the time of sampling using the Clauss assay by the FEISTY trial investigators [35].

### Plasmin generation

The rate of plasmin activation in plasma (10%) was measured using 0.5 mM S-2251 chromogenic substrate (Chromogenix, Ohio, USA) in the presence of 10 nM tPA (Actilyse, Boehringer Ingelheim, Germany). CBNr fibrinogen fragments (10 µg/ml;Technoclone, Vienna, Austria) were included to stimulate tPA activity. Absorbance readings at 405 nm were taken every 30 s for 8 h at 37 °C on a Biotek Flx800 microplate reader. The rate of plasmin generation was determined using Shiny App for zymogen activation rates [38].

### PAI-1 and FXIII activity assay

PAI-1 activity was measured using a commercial chromogenic assay following the manufacturer’s instructions (Molecular Innovations, Michigan, USA). FXIII activity in plasma was quantified using an in-house activity assay as previously described [39, 40].

### Confocal microscopy

Clots were formed from 30% plasma, 0.25 µM Alexa Fluor 488 (AF488) fibrinogen (ThermoFisher Scientific, Massachusetts, USA) and 16 µM phospholipids (Rossix, Molndal, Sweden). In some cases, an AF555 labelled monoclonal antibody against cross-linked fibrin (Zedira, Darmstadt, Germany) was incorporated. Clotting was initiated with 0.1 U/ml thrombin (Sigma-Aldrich, St Louis, USA) and 10.6 mM CaCl_2_ before adding to Ibidi µ-slide VI^0.4^ chambers (Ibidi GmbH, Gräfelfing, Germany). Patients from the cryo and Fg-C cohort were matched on their fibrinogen concentration in the pre-transfusion sample, so that cohorts were comparable. Representative images are shown in the manuscript. Images were recorded on Zeiss 880 laser scanning confocal microscope with a 63 X 1.40 oil immersion objective using Zeiss Zen 2012 SP1 software (black edition). Images were analysed using FIJI v1.51 and Diameter J plug in.

### Lateral atomic force microscopy

This method is described in detail by Duval et al. [41]. Briefly, plasma was diluted to obtain a fibrinogen concentration of 0.5 mg/ml and clotting initiated with 0.5 U/ml thrombin and 10.6 mM CaCl2. Clots were allowed to form for 1.5 h in a humid chamber prior to a washing step with tris buffered saline. Fibers were then incubated with 20-nm-yellow-green carboxylate FluoSpheres (Thermo Fisher Scientific) for 10 min, then washed again. A MFP3D atomic force microscope (Asylum Research, Oxford Instruments, Santa Barbara, CA) combined with an Axiovert 200 optical fluorescence microscope (Zeiss, Jena, Germany) were used to measure the mechanical response of individual fibrin fibres upon lateral stretching. Individual fibres were pulled by CSC38 cantilevers (MikroMasch, Tallinn, Estonia) until rupture, while the deformation was visualised with the fluorescence microscope. For each fibre a stress (calculated from the lateral deflection of the cantilever) vs strain (calculated from the position of the cantilever) curve was plotted and analysed to obtain a range of parameters to quantify fibre mechanical properties. In this work, we report on Modulus 1 (the fibre stiffness at low strain of 1.5) and toughness (the amount of energy required to rupture the fibre) parameters.

### Data analysis

Results are expressed as mean and standard deviation (SD). Grey dotted lines on graphs indicate the mean value for PNP control. Statistical analysis was performed using GraphPad Prism Software (v9.2.0; California, USA). Statistical significance pre- and post-fibrinogen replacement was determined using a paired *t*-test and *p* < 0.05 was considered significant. Correlations were analysed using Pearson correlation coefficients.

## Results

Plasma samples and accompanying clinical data were obtained pre- and post-fibrinogen transfusion from the FEISTY RCT investigators (Table 1). In this sub-study, we evaluated data from 9 cryo and 13 Fg-C patients who had comparable baseline admission characteristics (Table 1). Although the two groups had different GCS; 15 *vs.* 3 in the cryo and Fg-C cohort, respectively (Table 1), they had identical ISS (both 29) and the type of injury was comparable between groups (Table 1). FEISTY patients were categorised into 1 of 3 groups based on their type of injury and the percentage of patients in each group was similar between the cryo and Fg-C cohorts; 1) multiple injuries, burns or other (66.6 *vs.* 53.8%), 2) head and other associated injuries (22.2 *vs*. 23.1%) and 3) chest and/or abdominal injuries only (11.1 *vs.* 23.1%; Table 1). The time to first dose was significantly faster in patients receiving Fg-C when compared to cryo (48 *vs.*112 min, respectively, *p* < 0.05; Table 1).

Trauma patients in both the cryo and Fg-C arm had similar fibrinogen levels prior to fibrinogen supplementation (Table 1, 2). This was evident in both Clauss fibrinogen; 2.03 ± 0.8 *vs.* 1.8 ± 0.5 g/L cryo and fibrinogen antigen levels measured by ELISA; 1.6 ± 0.6 *vs.* 1.9 ± 0.5 mg/ml for the cryo and Fg-C groups, respectively (Table 1, 2). After transfusion with cryo, there was a 1.4- and 1.5-fold increase in Clauss fibrinogen and fibrinogen antigen, respectively (Table 2). Transfusion with Fg-C yielded a similar result; a 1.4- and 1.3-fold increase was observed in Clauss fibrinogen and fibrinogen antigen, respectively (Table 2). There was a weak positive correlation between fibrinogen antigen and Clauss fibrinogen levels both pre- and post-fibrinogen transfusion (*r*^2^ = 0.4 and 0.5, respectively). ROTEM analysis showed an increase in the clot amplitude at 5 min (CA5) and maximum clot firmness (MCF) in the FIBTEM, but not EXTEM, assay post-fibrinogen replacement with both cryo and Fg-C (*p* < 0.05, Additional file 1: Fig. S1). The trauma patients had elevated levels of the inflammatory markers, CRP and D-Dimer, as expected in this acute setting (Additional file 1: Fig. S2 A, B). Interestingly, we did not observe any changes in the thrombin generating potential of patients in the cryo and Fg-C cohorts after treatment, suggesting that dysfunction of fibrinolysis, and not coagulation, is key during trauma coagulopathy (data not shown).

**Table 2:** Fibrinolytic antigens levels. Fibrinogen, *α*_2_AP, TAFI, PAI-1, tPA, FXIII and thrombomodulin (TM) antigen concentration were measured in plasma samples pre- and post-cryoprecipitate (cryo) and fibrinogen concentrate (Fg-C) transfusion. Grey dotted lines indicate the mean value for 16 healthy volunteers. *p < 0.05, **p < 0.01

	Control	Cryoprecipitate			Fg-C		
		Pre	Post	*p*	Pre	Post	*p*
Fibrinogen (mg/ml)	3.5 ± 3.2	1.6 ± 0.6	2.3 ± 1.1	ns	1.9 ± 0.5	2.5 ± 0.8	ns
α_2_AP (µg/ml)	69.6 ± 27.2	33.6 ± 11	31.6 ± 12.2	ns	37 ± 10.4	31.6 ± 10.4	ns
TAFI (µg/ml)	8.1 ± 2	5.6 ± 2.4	5.3 ± 2.1	ns	5.5 ± 1.7	4.5 ± 1.8	*
PAI-1 (ng/ml)	11.7 ± 7.8	133.1 ± 159.7	278.3 ± 225.8	*	88.7 ± 81.9	93.7 ± 93.3	ns
tPA (ng/ml)	1.1 ± 0.5	3.5 ± 2.6	2.4 ± 1.3	ns	3.5 ± 1.7	3.8 ± 3	ns
FXIII (µg/ml)	39 ± 19.5	17.5 ± 3.8	22.5 ± 6.9	*	22.2 ± 7.2	18.3 ± 6.2	*
TM (ng/ml)	7.3 ± 1.7	9 ± 1.9	8.1 ± 2.3	ns	10.1 ± 6	9.6 ± 5.3	ns

We quantified a panel of fibrinolytic proteins in the patient groups and compared pre- and post-fibrinogen supplementation (Table 2). We found significantly lower concentrations of plasma *α*_2_AP in trauma patients prior to transfusion when compared to healthy controls; 33.6, 36.9 and 70.0 µg/ml in the cryo, Fg-C and healthy control groups, respectively (*p* < 0.0001; Table 2). Fibrinogen supplementation with cryo or Fg-C did not alter plasma α_2_AP concentration (Table 2), despite the fact that cryo was found to contain comparable levels of α_2_AP to PNP; 69.6 *vs.* 59.2 µg/ml, respectively (Additional file 1: Table S1). Similarly, we found that TAFI plasma levels were decreased in both cryo and Fg-C cohorts when compared to healthy controls; 5.6, 5.5 and 8.1 µg/ml, respectively (Table 2). Samples taken post Fg-C transfusion saw a significant 26.7% decrease in TAFI levels (*p* < 0.05, Table 2). There was no change in TAFI antigen after cryo transfusion (Table 2). Interestingly, there is more TAFI per ml cryo than plasma, suggesting cryo is supplementing the TAFI consumed during haemorrhage (Additional file 1: Table S1). In contrast, PAI-1 antigen was significantly elevated in all trauma patients prior to fibrinogen transfusion, observed as a 9- and 6-fold increase in the cryo and Fg-C groups; significantly higher than the healthy controls (14.4 ng/ml, *p* < 0.0001; Table 2). Fibrinogen supplementation with cryo further increased PAI-1 twofold (133.1 *vs.* 278.3 ng/ml pre- and post-transfusion); The concentration of PAI-1 antigen did not change post Fg-C transfusion (Table 2). A small decrease in tPA antigen (not significant) was also observed post-cryo transfusion (*p* = 0.05; Table 2). The mean concentration of tPA was unaltered pre- and post- Fg-C; 3.5 *vs*. 3.7 ng/ml, respectively (Table 2). However, tPA antigen levels were elevated in the trauma patients; 3.5, 3.5 and 1.1 ng/ml for cryo, Fg-C and healthy control cohorts, respectively (Table 2). Interestingly, uPA concentration was decreased in trauma patients at the time of sampling (Additional file 1: Fig. S2C). Together, these data suggest changes post-cryo transfusion were anti-fibrinolytic, whereas post-Fg-C changes would favour fibrinolysis.

Plasma levels of the transglutaminase FXIII were significantly reduced in both trauma cohorts; 17.5 and 22.2 µg/ml, respectively; when compared to healthy controls (39.0 µg/ml; Table 2). Interestingly, cryo and Fg-C transfusion produced contrasting effects. There was a significant increase in FXIII post-cryo transfusion (1.3-fold, *p* < 0.05), whereas a significant decrease was observed in the Fg-C cohort (0.8-fold, *p* < 0.05; Table 2). This is consistent with detection of FXIII in cryo at a higher concentration than PNP; 57.7 *vs.* 39.0 µg/ml, respectively (Additional file 1: Table S1), whereas minimal amounts were detected in Fg-C (0.4 µg/ml; Additional file 1: Table S1).

Levels of soluble TM were measured as a marker of endothelial activation (Table 2). We observed a significant increase in TM in both cohorts of trauma patients prior to fibrinogen transfusion when compared to healthy controls; 9.0, 10.1 and 7.3 ng/ml for the cryo, Fg-C and healthy control cohorts, respectively (*p* < 0.01, Table 2). A second endothelial marker, Syndecan-1, was also found to be elevated prior to fibrinogen transfusion (Table 2). Neither endothelial marker was affected by administration of either cryo or Fg-C.

**Fig. 1:**
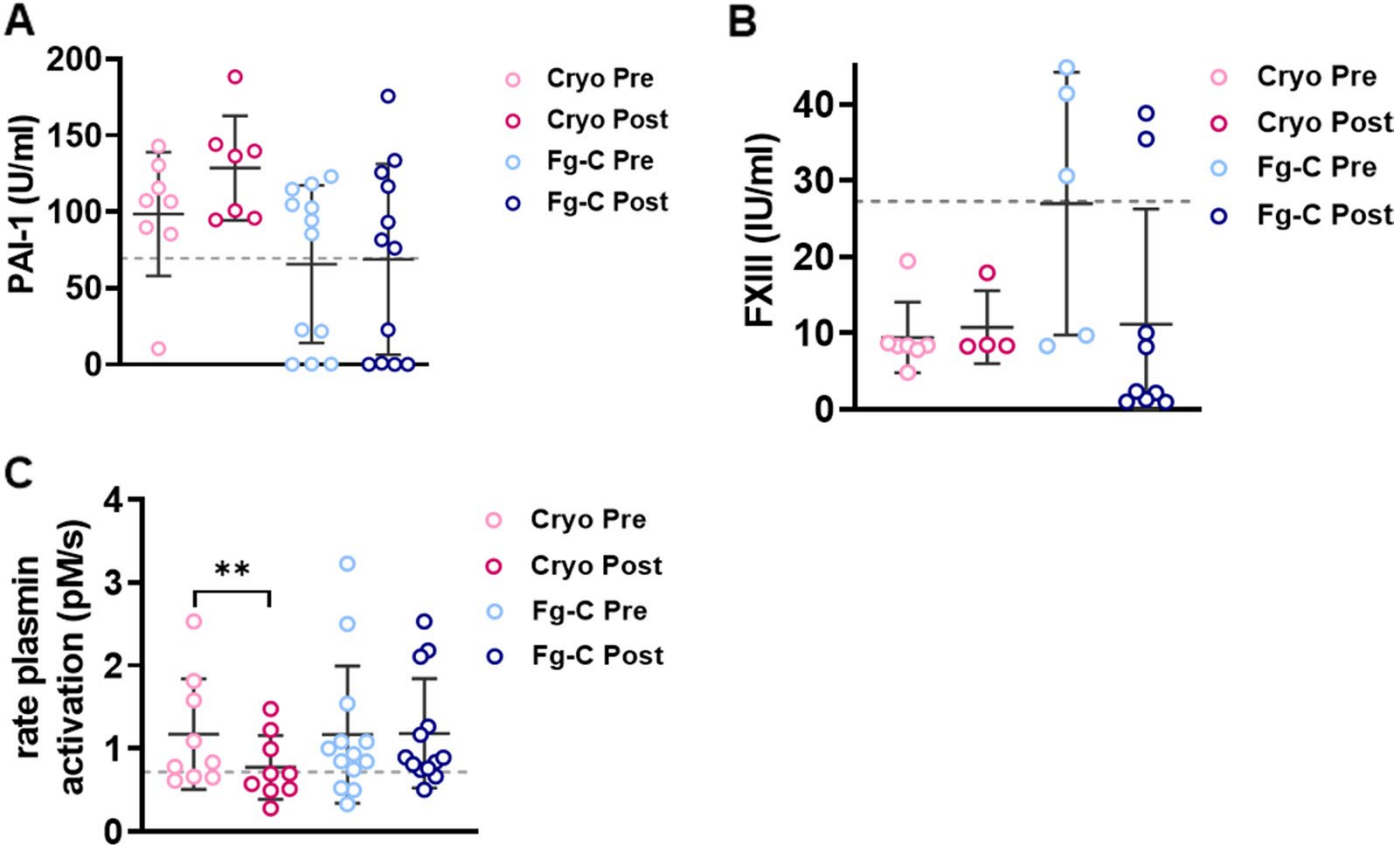
Cryoprecipitate transfusion attenuates fibrinolysis. **A** PAI-1, **B** FXIII and **C** plasmin activity levels were measured using chromogenic assays in plasma samples pre- and post-cryoprecipitate (cryo) and fibrinogen concentrate (Fg-C) transfusion. Grey dotted lines indicate the mean value for 16 healthy volunteers. **p < 0.01

To investigate how these changes in fibrinolytic antigens altered the fibrinolytic potential, we quantified plasmin generation alongside PAI-1 and FXIII activity. In agreement with the elevated levels of PAI-1 antigen, we found that PAI-1 activity was increased in the majority of trauma patients prior to fibrinogen supplementation (Fig. 1A). There was a strong positive correlation between PAI-1 antigen and activity levels (*r*^2^ = 0.7, *p* < 0.001, data not shown). Interestingly, we observed an increase in PAI-1 activity in the cryo cohort; 98.7 *vs.* 128.7 U/ml pre- and post-cryo transfusion, respectively (Fig. 1A). The mean PAI-1 activity level remained constant pre- and post-Fg-C transfusion; 65.8 *vs.* 69.6 U/ml (Fig. 1A). In agreement with the FXIII antigen results, analysis of FXIII activity in plasma samples revealed trauma patients in the cryo group had significantly reduced FXIII activity (Fig. 1B). Patients in the Fg-C cohort had FXIII levels similar to the healthy controls (Fig. 1B). Therefore, we opted to measure the rate of plasmin activation using an in-house plasmin generation assay, which confirmed significantly elevated levels of plasmin generation in trauma patients when compared to healthy controls (*p* < 0.01, Fig. 1C). Fibrinogen supplementation with cryo, but not Fg–C, resulted in a decrease in the amount of plasmin generated (Fig. 1C). This was observed as a 1.5-fold decrease in the rate of plasmin generation post-cryo transfusion (*p* < 0.01; Fig. 1C).

**Fig. 2:**
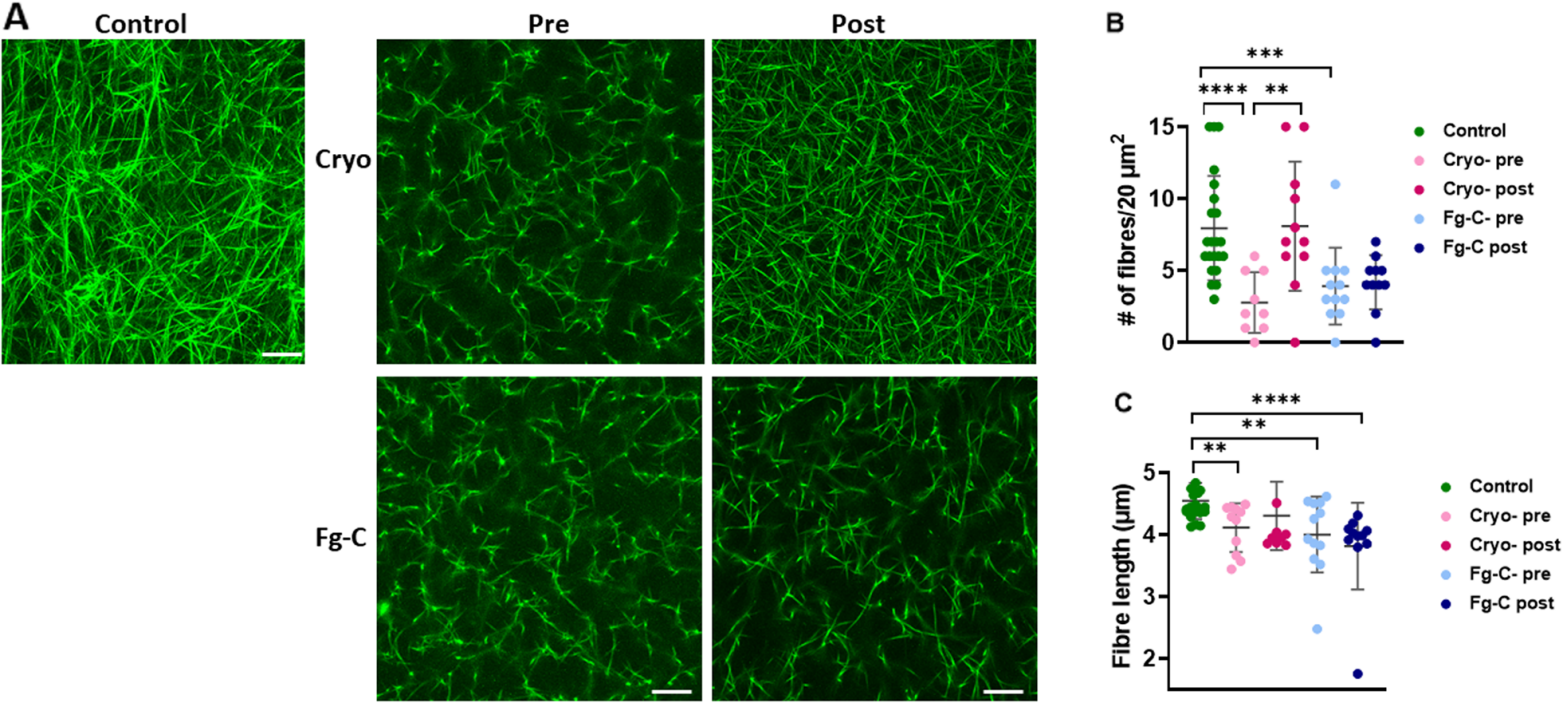
Clots formed post cryoprecipitate have increased fibre density. **A** Plasma clots were formed from pooled normal plasma (PNP) or patient plasma pre- and post-cryoprecipitate (cryo) or fibrinogen concentrate (Fg-C) transfusion. Alexa Fluor 488 labelled fibrinogen was incorporated to visualise the fibrin network by confocal microscopy. Representative images of *n* = 3 per cohort. Clots were formed in duplicate. **B** number of fibres and **C** fibre length were calculated using Diameter J software. Three areas were analysed per clot. **p < 0.01, ***p < 0.001, ****p < 0.0001

Next, we investigated the effects of cryo and Fg-C supplementation on the fibrin network. Plasma clots formed in the presence of AF488 fibrinogen were analysed using confocal microscopy (Fig. 2A). Clots formed from trauma patients across both cryo and Fg-C groups had a significantly lower number of fibres per 20 µm^2^ when compared to control clots (*p* < 0.001, Fig. 2B). Visually, the fibrin network was more porous and the fibres appeared stunted in clots formed from trauma patient plasma, compared to the PNP control (Fig. 2A). This was confirmed by Diameter J analysis; revealing trauma patients had a significantly shorter fibre length; 4.1, 4.0 and 4.5 µm in the cryo, Fg–C and healthy control cohorts, respectively (*p* < 0.001, Fig. 2C). Interestingly, clots formed from samples taken post-cryo transfusion were visually different to those taken pre transfusion and the fibrin network appeared denser (Fig. 2A). This was confirmed in the statistical analysis, which found no significant difference in the number of fibres or fibre length between clots formed from post-cryo plasma and PNP (Fig. 2B, C). In contrast, the abnormal fibrin network persisted post Fg–C transfusion and the clots remained porous with stunted fibres (Fig. 2A). Neither the fibre length nor the number of fibres was altered post Fg-C transfusion in the statistical analysis (Figs. 2B, C).

**Fig. 3:**
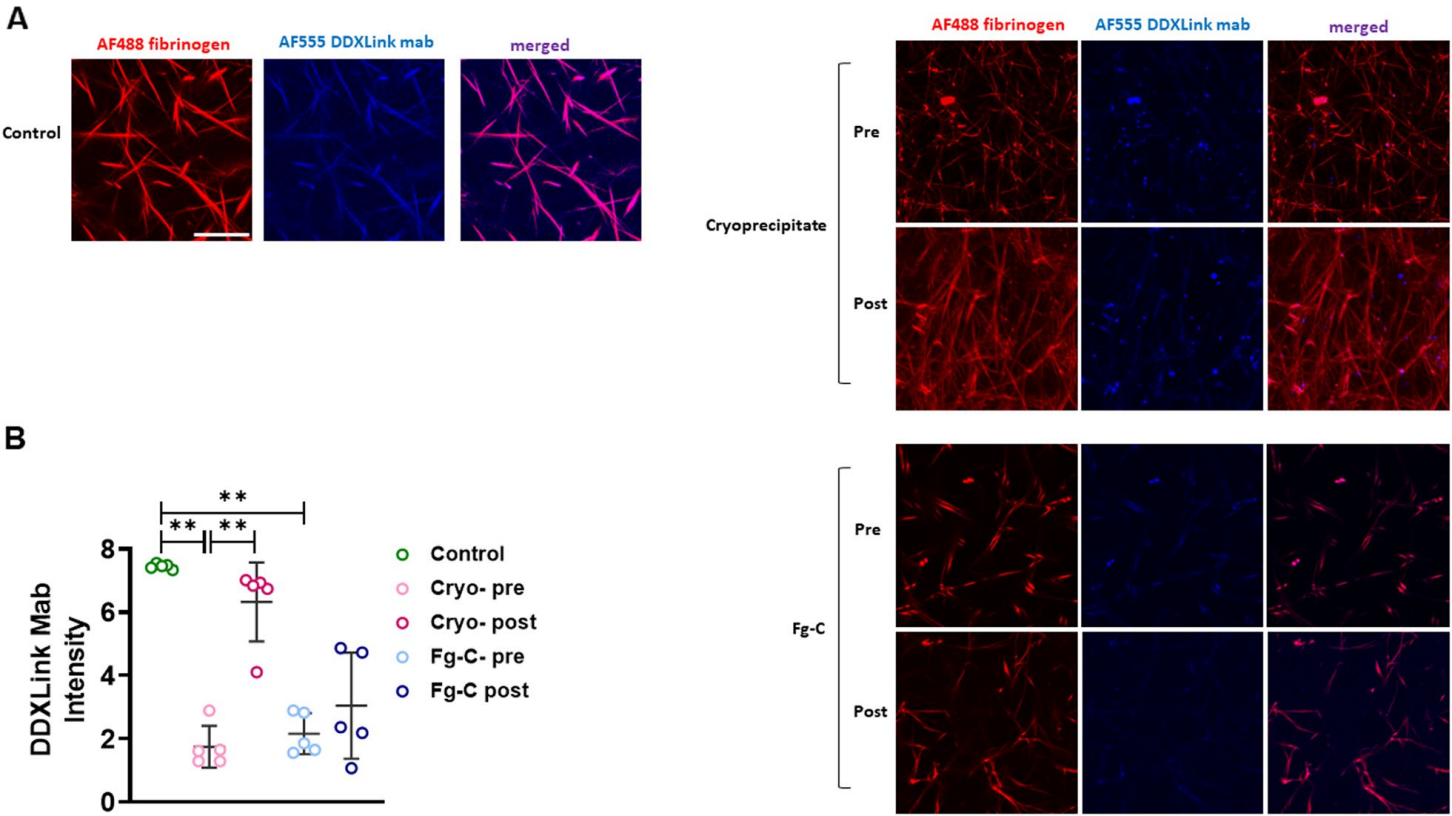
Factor XIII cross-linking is attenuated in trauma patients. **A** Plasma clots were formed from pooled normal plasma (PNP) or patient plasma pre- and post-cryoprecipitate (cryo) or fibrinogen concentrate (Fg-C) transfusion. Alexa Fluor 488 labelled fibrinogen (red) and Alexa Fluor 555 DDXLink monoclonal antibody (blue) specific for fibrin cross-links was incorporated to visualise the fibrin network by confocal microscopy. Representative images of *n* = 3 per cohort. **B** The intensity of DDXLink monoclonal antibody staining was determined using Image J software. Three areas were analysed per clot. **p < 0.01

To further investigate the stability of the fibrin network in trauma patients we used an AF555 fluorescently labelled antibody specific for cross-linked fibrin (DDXLink mab; Fig. 3). In clots formed from PNP, fibrin cross-links were abundant and were linearly distributed along fibrin fibres with AF488 fibrinogen staining (Fig. 3A). In clots formed from trauma patients, the number of fibrin cross-links appeared visibly less and were not present over the full length of the fibrin strands (Fig. 3A). Quantification of AF555 DDXLink mab staining intensity demonstrated a significant decrease in the brightness of fibrin cross-link staining in both the Fg-C and cryo groups pre transfusion when compared to control clots (*p* < 0.01, Fig. 3B). Interestingly, a significant increase in the intensity of DDXLink mab staining was observed in clots formed from plasma post-cryo transfusion (*p* < 0.01, Fig. 3B). No change in intensity was observed in the Fg-C cohort (Fig. 3B).

**Fig. 4:**
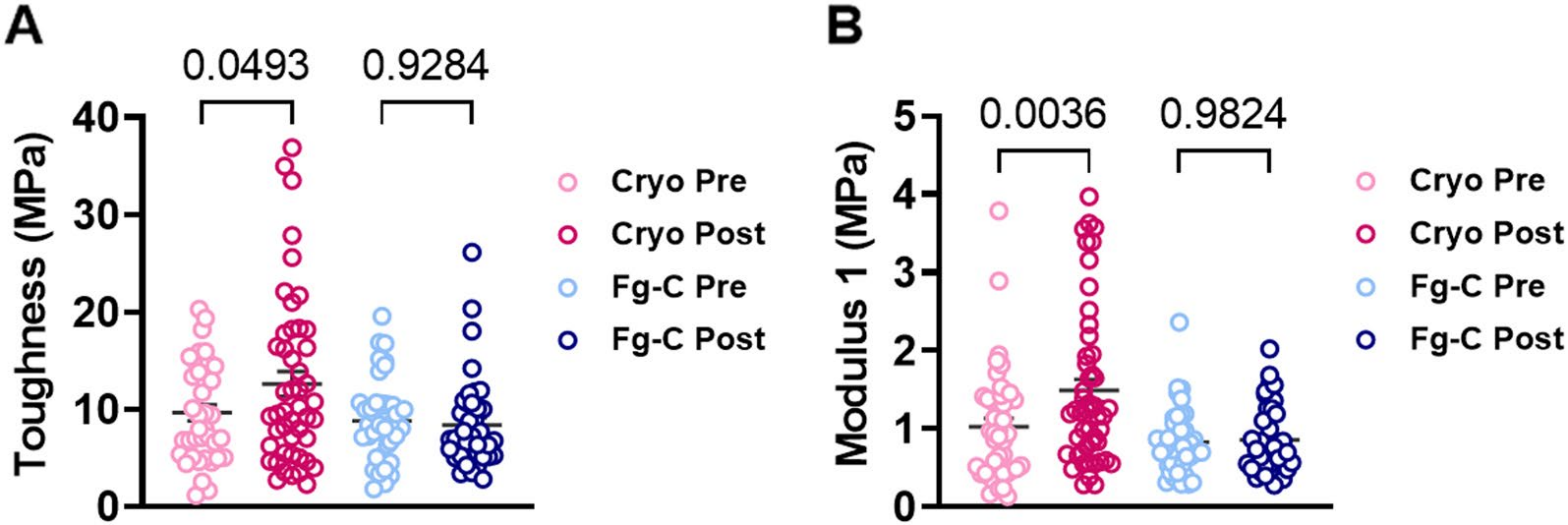
Cryoprecipitate transfusion augments fibrin fibres strength. The mechanical response of individual fibrin fibres in plasma clots formed pre- and post-cryoprecipitate (cryo) and fibrinogen concentrate (Fg-C) were analysed using atomic force microscopy and **A** toughness and **B** Modulus 1 calculated

To assess how these changes in the fibrin network altered individual fibre strength, we utilised atomic force microscopy to determine the mechanical response of individual fibrin fibres upon lateral stretching (Fig. 4A). We found that cryo, but not Fg-C, transfusion resulted in a significant increase in the toughness of the fibrin fibres (*p* < 0.05, Fig. 4A). The toughness is the amount of energy required to rupture the fibre, therefore fibres formed in the presence of cryo showed higher resistance to rupture than those formed from Fg-C. We also found that the fibres displayed an increase in Modulus 1 (the stiffness of the fibres under a low strain of 1.5) in the post-cryo cohort when compared to the pre transfusion group (*p* < 0.01, Fig. 4B). Fg-C did not alter fibre stiffness at low strain.

## Discussion

Traumatic coagulopathy is characterised by low fibrinogen and hyperfibrinolysis. Fibrinogen is indispensable for stable blood clot formation, therefore normal haemostasis is dependent on fibrinogen levels [42, 43]. This study uses plasma samples obtained from the FEISTY trial; the first trial to compare cryo and Fg-C in a trauma RCT setting; with the primary outcome of whether fibrinogen replacement to reverse hypofibrinogenaemia could be achieved faster with Fg-C [36]. Our data provide highly relevant information about the differences between these transfusion products at the molecular level of the clot itself. We reveal that the ex vivo rate of fibrinolysis is decreased in patients following cryo, but not Fg-C, transfusion. This was evident as a decrease in the rate of plasmin activation consistent with the documented increase in PAI-1 antigen and activity in the cryo cohort. Furthermore, confocal analysis of fibrin clot structure revealed a more homogenous and less porous structure in clots formed from cryoprecipitate patient plasma, but not for the Fg-C. Parallel with this we found that cryo, but not Fg-C, transfusion resulted in a significant increase in the toughness of individual fibrin fibres. This is consistent with an increase in FXIII antigen in the cryo cohort and likely mediated by the increased in fibre cross linking.

While fibrinogen is the first coagulation protein to be depleted during massive haemorrhage [17, 44], there is limited evidence to support a specific fibrinogen concentration that should be targeted to manage active bleeding [45]. Fibrinogen can be replaced using fresh frozen plasma (FFP), cryo, or Fg-C, all of which have varying concentrations of fibrinogen (2 g/L, 8–16 g/L and 20 g/L, respectively). Both Fg-C and cryo have high concentrations of fibrinogen and have various benefits and disadvantages. The low concentrations of fibrinogen in FFP make it unsuitable for fibrinogen supplementation [46, 47], and the efficacy of either cryo or Fg-C in major trauma haemorrhage remains undetermined by a RCT. CRYOSTAT-2, a RCT addressing whether early cryo transfusion improves survival from major trauma haemorrhage, will address this question. It has finished recruiting patients and the forthcoming results are eagerly awaited [30]. If CRYOSTAT-2 shows that early fibrinogen supplementation is effective, the comparative effects of Fg-C and cryo are highly relevant. Our study begins to bridge this knowledge gap and will be further investigated by the FEISTY-2 trial which will compare Fg-C and cryo in 800 trauma patients across Australia and New Zealand. Previously, the FIBRES trial, an RCT comparing Fg-C and cryo in cardiac surgery found that Fg-C was non-inferior to cryo based on an outcome measure of blood products transfused 24 h post bypass [48]. From a clinical perspective, it is important to note that not all replacement therapies for fibrinogen have identical composition [33, 34], and the authors would suggest that these types of analysis should be extended to all types of fibrinogen concentrate on the market.

One of our main findings is the attenuation of plasmin generation after cryo but not Fg-C transfusion. To minimise the known effect of fibrinogen concentration on the rate of plasmin generation [43], we compared equivalent concentrations of fibrinogen between the two patient cohorts; 2 *vs.* 1.9 mg/ml for the cryo and Fg-C groups, respectively. Our data reveal a decrease in the rate of plasmin activation in the cryo group which is plausibly explained by our antigen data showing increased PAI-1 and decreased tPA. These changes in fibrinolytic antigens and plasmin generating potential were not observed in the Fg-C group. The combined effect of dampening hyperfibrinolysis while replacing fibrinogen is likely to be of benefit in trauma patients.

The concentration of TAFI, which inhibits fibrinolysis by removal of the C-terminal lysine residues from fibrin, decreased in the Fg-C group, suggesting cryo may be supplementing TAFI levels that are depleted during major haemorrhage. Although α_2_AP concentration was not altered by cryo or Fg-C transfusion, cryo does contain significant amounts of α_2_AP, whereas no α_2_AP was detected in Fg-C, suggesting the *α*_2_AP present in cryo may be rapidly cross-linked to fibrin or complexed with plasmin. The low levels of *α*_2_AP detected in the trauma patients are in agreement with the significantly elevated levels of plasmin being generated.

Perhaps most striking is the confocal microscopy analysis of clots formed from trauma patients. Previously, we have shown clots formed from fibrinogen deficient plasma spiked with cryo or Fg-C have structural differences [37]. Here, clots formed from patient plasma, taken during active bleeding and pre- and post-fibrinogen supplementation, confirm our in vitro observations. Clots formed following cryo transfusion exhibit a homogeneous fibrin network, similar to those formed from control plasma. The clots formed from patient samples after Fg-C transfusion remained porous with stunted fibres. Fibrin composition is known to affect the rate of fibrinolysis [45–49]; the size, number and arrangement of fibrin fibres can influence the rate of fibrinolysis as they determine the extent of tPA binding to fibrin [49–53]. The porosity of the clot also influences its susceptibility to fibrinolysis; clots which have a dense fibrin network hinder the diffusion of fibrinolytic enzymes, whereas porous clots facilitate the movement of the degradation agents [49, 54]. Therefore, the porous clots with stunted fibres formed by patients treated with Fg-C will be more susceptible to fibrinolytic degradation than the homogeneous fibrin network that forms in clots of patients who were treated with cryoprecipitate.

Coagulation FXIII plays a critical role in forming fibrin-fibrin and fibrin-*α*_2_AP cross-links [21–23], therefore we hypothesised that the abundance of FXIII present in cryo may alter the biomechanical strength and resistance to fibrinolysis structure of the fibrin network. Interestingly, staining plasma clots with a monoclonal antibody specific for fibrin cross-links revealed an increase in cross-links post-cryo, but not Fg-C, transfusion. Similarly, we found that FXIII antigen concentration was significantly increased post-cryo transfusion and, conversely, significantly decreased post Fg-C concentration. FXIII activity was lower in the cryo cohort, but normal in the Fg-C group pre-transfusion. No recovery was observed post-transfusion, with the Fg-C cohort activity levels decreasing. To evaluate the effects of FXIII in a more physiologically relevant setting, we utilised atomic force microscopy to measure the toughness of individual fibrin fibres. Our analysis confirmed that fibres formed from the post-cryo cohort required more energy to rupture than their Fg-C counterparts. These data suggest clots formed post-cryo transfusion are more stable than those formed post Fg-C. The increased strength of fibrin fibres is likely due to the increased FXIII concentration observed in cryo, as FXIII cross-linking enhances the tensile and flexural stiffness of individual fibres [41, 55–57]. Previous in vitro studies using a ROTEM model of dilutional coagulopathy have shown that combined fibrinogen and FXIII concentrates are highly effective at increasing the maximum clot firmness [58, 59]. However, it is unknown how ROTEM parameters relate to biophysical measures of viscoelasticity such as stiffness and elastic modulus. Our data on fibre toughness and modulus 1 indicate that these measures are specifically improved by cryo supplementation. This warrants further investigation to determine if there is a rationale for combining fibrinogen supplementation using cryo or Fg-C with FXIII concentrate in managing trauma coagulopathy. The Fg-C used in this study (Riastap) does not contain FXIII but recent in vitro studies evaluating different Fg-C preparations has shown that those containing FXIII perform similarly to cryoprecipitate in measures of clot strength and stability [33, 34]. Our data combined with these in vitro studies suggest using a Fg-C that contains FXIII would be superior to using one without FXIII.

Although cryo and fibrinogen both provide ample concentrations of fibrinogen, cryo contains a number of coagulation and fibrinolytic factors that are not present in Fg-C (Additional file 1: Table S1) [31]. Fg-C has advantages including standard dose per vial, ease of transport and reduced transfusion volume, however it does undergo a number of processing steps that may alter its properties (heat treatment, glycine precipitation and lyophilisation). The benefits of Fg-C preparation are demonstrated by the time to first dose, however, the time to haemostasis was similar for both patient groups; although this was not collected for all patients. Importantly, the development of single factor concentrate therapies, such as Fg-C and factor VIII concentrates, have phased out cryo use as a first line therapy for heritable bleeding disorders [60]. Our study has shown that fibrinogen supplementation with cryo has additional benefits over Fg-C and has highlighted that there should be re-evaluation of the global phasing out of cryo production or development of multi-factor concentrates.

There are limitations to our study, most notably the small patient number. Despite this, we have presented data that show clear physiological differences in clot formation between two groups receiving different forms of fibrinogen supplementation. The small groups result in some differences in baseline clinical data; for example there were more patients with a low GCS in the Fg-C group but the number of patients with significant TBI (AIS 2 or more) was comparable (*n* = 3 in Fg-C and *n* = 2 in cryo arms). Another crucial difference is that 53.8% of Fg-C patients received TXA, whereas only 22.2% of cryo patients received TXA. Theoretically, we would expect that this would lead to reduced fibrinolysis in the Fg-C group, however, the Fg-C group demonstrated more porous clots that were more susceptible to fibrinolysis, in comparison to the cryo patients. This may be due to the fact that TXA was administered pre-hospital and some of the drug may have been eliminated by the time of sampling. Furthermore, we would not expect TXA to impact fibrin structure or the majority of the antigen or activity results for PAI-1 or FXIII.

## Conclusions

We have shown that cryo supplementation reduced susceptibility to fibrinolysis, demonstrated by the attenuation of plasmin activation, an increase in PAI-1 and maintenance of TAFI. Cryo clots displayed a more homogenous fibrin network with an increased number of fibres than those formed post Fg-C. Analysis of FXIII and individual fibrin fibres indicated that these clots were composed of fibres that were more resistant to mechanical disruption. Taken together, our data suggest that clots formed following fibrinogen supplementation with cryo are more extensive, physically stronger and more resistant to fibrinolytic degradation. These differences may translate into alterations in efficacy and clinical outcomes for trauma patients, which will be tested in FEISTY-2 RCT.

## Supplementary Information

**Additional file 1**. Supplementary Figures.

## Data Availability

For original data, please contact gael.morrow@ndcls.ox.ac.uk.
