# Supplementary material for: Cryoprecipitate transfusion in trauma patients attenuates hyperfibrinolysis and restores normal clot structure and stability: Results from a laboratory sub-study of the FEISTY trial
Source: Crit Care. 2022 Sep 26;26:290. doi: 10.1186/s13054-022-04167-x (PMC9511733; doi:10.1186/s13054-022-04167-x)
Supplement: Supplementary file 1 — Additional file 1. Supplementary Figures. [file 13054_2022_4167_MOESM1_ESM.docx]

**Supplementary Data**

| **Antigen** | **PNP** | **Cryoprecipitate** | **Fibrinogen concentrate** |
| --- | --- | --- | --- |
| Fibrinogen (mg/ml) | 4.2 ± 0.6 | 7.4 ± 0.7 | 21.6 ± 0.1 |
| α2AP (µg/ml) | 69.6 ± 27.2 | 59.2 ± 3.3 | 0 |
| FXIII (µg/ml) | 39 ± 19.5 | 57.7 ± 28.8 | 0.4 ± 0.02 |
| PAI-1 (ng/ml) | 9.6 ± 8.3 | 8.2 ± 0.2 | 1 ± 0.1 |
| TAFI (µg/ml) | 8.1 ± 2.0 | 15.0 ± 6.4 | 0.3 ± 0.3 |
| TM (ng/ml) | 7.3 ± 1.7 | 10.7 ± 2.2 | 1.0 ± 0.9 |
| Syndecan-1 (ng/ml) | 40.1 ± | 66.8 ± 26 | 0 |
| tPA (ng/ml) | 1.1 ± 0.5 | 3.4 ± 0.3 | 0.3 ± 0.2 |
| uPA (ng/ml) | 1.3 ± 0.01 | 0.6 ± 0.1 | 0 |

***Table S1. Concentrations of fibrinolytic proteins in cryoprecipitate and fibrinogen concentrate.*** Plasma antigen levels for a panel of fibrinolytic proteins were measured in pooled normal plasma (PNP), cryoprecipitate and Fg-C. Data expressed at mean ± SD.

***Figure S1. FIBTEM, but not EXTEM, measures of clot firmness are increased post-fibrinogen transfusion.*** ROTEM was performed on whole blood samples from FEISTY trauma patients pre- and post- transfusion with cryoprecipitate (cryo) or fibrinogen concentrate (Fg-C). The (A) clot amplitude at 5 minutes (CA5) and (B) maximum clot firmness (MCF) for FIBTEM; which measures the contribution of fibrinogen to the clot. The (C) CA5 and (D) MCF were also obtained for EXTEM; which determines the contribution of the extrinsic pathway. * *p* < 0.05, ** *p* < 0.01, *** *p* < 0.001.

***Figure S2.*** ***Inflammatory and endothelial markers are elevated in trauma patients.*** (A) CRP, (B) D-Dimer, (C) uPA and (D) Syndecan-1 antigen concentration were measured in plasma samples pre- and post- cryoprecipitate (cryo) and fibrinogen concentrate (Fg-C) transfusion. Grey dotted lines indicate the normal range from 16 healthy volunteers. * *p* < 0.05. ** *p* < 0.01 *vs.* healthy controls.
